# Nonpolyp‐associated otitis media in cats: The little we know

**DOI:** 10.1002/vms3.850

**Published:** 2022-06-11

**Authors:** Mariana Bezerra Mascarenhas

**Affiliations:** ^1^ Dermatology Service Promove Clínica Veterinária Rio de Janeiro Brazil

Otitis media (OM) is characterised by inflammation and infection in the middle ear. Despite being a common condition in cats, the pathogenesis of OM is not well understood. The known causes of middle ear disease (MED) in cats are inflammatory polyps, neoplasia and extension of external ear canal infection into the middle ear (Shanaman et al., 2011; Swales et al., 2018). Auditory tube obstruction or dysfunction secondary to nasal or upper respiratory disease (Detweiler et al., 2006; Shanaman et al., 2011) and rarely haematogenous spread (Shanaman et al., 2011) are suggested causes of MED with effusion.

The association between MED and nasal disease on computed tomography (CT) scans in cats has been documented previously (Detweiler et al., 2006; Shanaman et al., 2011). The prevalence of bulla effusion in cats with sinonasal disease was 28% (13/46) and was significantly higher than that observed in cats without sinonasal disease (1/18; Detweiler et al., 2006). In another study, 27 of 34 cats (79%) had concurrent nasal disease (Shanaman et al., 2011). Experimental obstruction of the auditory tube causes a decrease in the middle ear ciliary area, decreased secretion clearance and middle ear effusion (Jin et al., 1991). This model potentially mimics some nasal and upper respiratory diseases in which mucosal inflammation and temporary auditory tube obstruction result in middle ear effusion. Viral upper respiratory tract infections may predispose patients to bacterial colonisation of the nasopharynx and ascending bacterial middle ear infection via the auditory tube (Detweiler et al., 2006). For those cats, recurrence of OM can occur if the nasal or upper respiratory disease is not well controlled. As most of the time, viral infections are the primary cause of these upper respiratory diseases in cats, adequate control of the upper respiratory disease is difficult. In the previously mentioned study (Detweiler et al., 2006), eight of the 25 cats with rhinitis had concurrent OM, including 2/2 cats with lymphoplasmacytic rhinitis, 4/14 with neutrophilic rhinitis and 2/8 with mixed inflammatory rhinitis. CT evidence of sinusitis and nasopharyngeal disease was positively correlated with the finding of OM. The implications of these findings for the management of cats with sinonasal disease require further investigation (Detweiler et al., 2006).

In a study of imaging evaluation of the middle ear, five of 84 cats (6%) were excluded as normal controls because they had fluid in the tympanic bulla (King et al., 2007). Another study showed that 34 of 101 cats (34%) did not have clinical signs of OM or physical findings consistent with ear disease, suggesting that MED was subclinical (Shanaman et al., 2011). Subclinical MED was relatively frequent in cats undergoing CT imaging of the head (Jin et al., 1991; Shanaman et al., 2011). Few cats had subsequent treatment for ear disease, suggesting that treatment may not be necessary, although follow‐up was limited in this study (Shanaman et al., 2011). At this time, it is not known if treatment is important for these cats and if asymptomatic OM can progress to become symptomatic. Chronic OM with effusion is thought to be associated with chronic inflammatory changes in the middle ear leading to abnormal fluid secretion by the membrane lining the tympanic bullae and auditory canal (Detweiler et al., 2006).

The percentage of cats that present with symptomatic OM without otitis externa (OE) and upper respiratory signs is lacking in the literature. In a study that evaluated 16 cats with OM, half of the cats showed signs of OE on presentation, and three cats presented with upper respiratory signs, while the other five cats (31%) presented symptomatic OM without OE and/or upper respiratory signs (Swales et al., 2018). The auditory tube dysfunction could be a potential cause as well as haematogenous spread or viral infections; however, further studies are needed.

OM can either be managed medically or surgically (Swales et al., 2018). Since the cause is often unknown, deciding on the ideal treatment protocol can be challenging in some cases. Myringotomy followed by middle ear flushing and therapy with glucocorticoids and antibiotics, if bacterial infection is present, have been described as a safe and efficient treatment modality for suppurative OM in cats (Deleporte & Prelaud, 2021; Swales et al., 2018). Although recurrence can occur, surgical procedures, such as ventral bulla osteotomy (Moore et al., 2019; Swales et al., 2018), have been performed in some cases, and tympanostomy tube placement (Lucas et al., 2020) has been tried to reduce the recurrence rate and as the treatment of OM with effusion.

Further studies are needed to evaluate the pathogenesis of OM in cats for a better treatment and control of the disease. Many questions remain unanswered. What is the importance of the subclinical MED? Do subclinical MED progress to symptomatic disease? Does subclinical OM need to be treated? Does viral infection of the upper respiratory tract contribute to the development of OM in cats? How can we manage cats with sinonasal disease to avoid chronicity and recurrence?

## CONFLICT OF INTEREST

The author declares no conflicts of interest.

## FUNDING INFORMATION

This study was self‐funded.

## ETHICS STATEMENT

The author confirms that the ethical policies of the journal, as noted on the journal's author guidelines page, have been adhered to. No ethical approval was required as this is a ‘Commentaries’ article with no original research data.

## AUTHOR CONTRIBUTION

*Conceptualisation, writing—original draft*: Mariana Bezerra Mascarenhas.

### PEER REVIEW

The peer review history for this article is available at https://publons.com/publon/10.1002/vms3.850

## Data Availability

Data availability are available from the corresponding author.
